# Supplementary material for: The impact origin and evolution of Chryse Planitia on Mars revealed by buried craters
Source: Nat Commun. 2019 Sep 18;10:4257. doi: 10.1038/s41467-019-12162-0 (PMC6751168; doi:10.1038/s41467-019-12162-0)
Supplement: Supplementary file 1 — Supplementary information [file 41467_2019_12162_MOESM1_ESM.doc]

Supplementary Information for

The impact origin and evolution of Chryse Planitia on Mars revealed by buried craters

by Pan et al.

This file includes 3 parts:

1. Supplementary Notes 1-6
2. Supplementary Figures 1-5
3. Supplementary Tables 1-3

*Supplementary Note 1 -The effect of erosion and burial*

Crater modifications take place on Mars via various processes, which could be generally classified into erosion, burial, and relaxation. Fluvial and aeolian erosions cause the topography profile to evolve analogous to a viscous relaxation process, resulting in a reduced depth-to-diameter ratio^1-5^. Fluvial erosion induces retreat of the crater wall, resulting in a reduced wall slope, and an enhanced elevation of the crater floor by infill^1,2^. In the case of burial, however, the wall slopes are better preserved, and the crater floor is quickly elevated forming a flat floor morphology with crater ejecta completely buried (Supplementary Figure 1). The variations of crater morphology on Mars cannot be accounted for with only one process. Within the Chryse and Acidalia region however, most of the impact craters with small depth-to-diameter ratios exhibit a flat-floored morphology with a raised rim. Although it is likely that these impacts experienced a certain degree of erosion, the main reason for shallowing the crater is due to a burial process, as shown by previous studies^6-8^. The bimodal distribution of depth-to-diameter ratio also supports the fact that the morphology cannot be due to erosional processes alone, given that erosional processes affect small craters preferentially and would result in a unimodal distribution of depth-to-diameter ratio.

*Supplementary Note 2 -The trade-off between obliteration and resurfacing*

We have explored various combinations of obliteration rate and resurfacing event to understand how well the model captures the impact crater population in the observed data. We find that although the bimodal distribution could be well represented for a one-time resurfacing event, the absolute values of depth-to-diameter are higher than what is observed if no obliteration is considered (Figure 3). It is also evident that shallow and small impact craters (D<7km) that would be completely removed in a large-scale (>100m) resurfacing event are present in the data, indicating a long-term or late resurfacing process after a resurfacing event at 3.7 Ga. To better evaluate the contributions of the resurfacing event with the presence of a constant obliteration rate, we test if the models are reproducing the observed crater population by calculating a chi-square statistic for impact craters (D>7km) in the degraded category (d/D < 0.04) (Method, Crater accumulation and modification; Supplementary Figure 2) with an obliteration rate of 0-100 m/Gyr and a fill thickness between 0 and 1km. Overall a larger fill thickness is preferred for Chryse than Adjacent unit for the same obliteration rate. At higher obliteration rate, the trade-off between obliteration rate and fill thickness is in control of the buried crater population and that all fill thicknesses may be allowed if obliteration rate can be as high as 80-100 m/Gyr.

Alternatively, the depletion of small craters resulting in a cumulative crater frequency crossing isochrons for both types of units could be interpreted as a secondary resurfacing event at 3.4-3.5 Ga (Figure 3a). The secondary event may be largely responsible for the small crater population in Adjacent unit but only partially affected Chryse anomaly unit. The different crater populations in Chryse and Adjacent units are thus a combination of the resurfacing event at 3.7 Ga, constant obliteration of small craters, and possibly a secondary event in Adjacent unit.

*Supplementary Note 3 -The thickness estimation for completely buried impact craters.*

We calculate the thickness of the overlying unit on completely buried craters assuming the excess fill is small. The possibility that completely buried impact craters may still be observed when covered by a thick lava flow or sediments (with a non-negligible excess fill) cannot be fully rejected if the overlying lava/sediment has compacted/contracted following drying/cooling.

Assuming all the overlying units compacts equally with a ratio $\alpha$ determined by the compaction property of the overlying unit, we find that the observed depth of the crater is proportional to the original depth, which is unrelated to its thickness. In this case, the observed depth of impacts ($d_{obs}$) would be:

$d_{obs} =(T_{rim}- T_{center})*\alpha=d_{0}*\alpha$ (0< $\alpha$ <1).

However, if there are secondary events and the compaction ratio stays the same, the resulting depth from these events would be:

$d_{obs} = d_{0}*\alpha^{n}$

*n* is the number of events where infill and deflation occurred. Therefore, if multiple events of infill and deflation occurred, this would largely reduce the surficial topographic expression. This means we cannot uniquely determine the thickness of the overlying unit, except that it is thicker than the original rim height (as estimated assuming minimal excess fill), i.e. the relationship between depth and diameter would be unrelated with infill thickness.

In contrast to a uniform deflation scenario, if the lava flow deflates differentially^9^ so that the compaction ratio varies with thickness, the depth of the observed depression resulting from compaction may be correlated with the thickness of fill. The relative deflation theory has intrinsic uncertainties since the origin of differential contraction is unclear and the differential deformation rate, which depends on material properties, has not been characterized. In this case, the thickness estimate under zero excess fill assumption would give a lower limit if we consider the compaction of the overlying unit. Although the excess overlying fill may be possible, there is no strong argument for differential compaction, and the parameterization for the compaction process is unknown.

*Supplementary Note 4 -The gravity signature of Chryse Planitia*

Within the circular Bouguer anomaly in the Chryse region, the positive gravity signature was muted and largely heterogeneous (Supplementary Figure 3a-b). This gravity signature contrasts with most large basin-forming impacts where strong positive gravity anomalies are found, lending the impact origin of Chryse an unresolved issue^­10,11^. Here we test the possibility of a lava infill event alone created the positive anomaly in the Chryse anomaly unit. Assuming the dense lava flow units have a density of 3300 kg/m^3^ and the underlying basement has an average of density of 2700~3300 kg/m^3^, we calculate the gravity anomaly assuming an infinite plateau of dense material with a density difference from 0 to 600 kg/m^3^. The difference in gravity anomaly is predicted to be:

$$\Delta G_{bouguer}= 2\pi*G*\Delta\rho*h$$

The resulting differences (Supplementary Figure 3c) is less than 30 mgal for the entire density variation range, indicating the gravity anomaly is not the result of the infilling of dense materials, but rather dominated by an uplifted mantle-crust boundary (thinning of crust), similar to lunar mascon basins^12,13^.

The gravity anomaly that requires mantle uplift supports the impact origin of the Chryse basin. In the meantime, heterogeneity of gravity signals within the Chryse unit could be due to various subsurface sedimentary units, since there are multiple outflow channels debouching into the basin^14,15^. In particular, the western part of the anomaly unit (which coincides with the termini of Kasei Valles outflow channel) shows slightly smaller Bouguer anomaly than the eastern part, likely a result of a more recent sedimentation event. The muted signal of gravity anomaly within the Chryse basin should be interpreted as a combination of the impact forming event and the complicated resurfacing history that followed.

*Supplementary Note 5 - Crater initial morphometry*

We assume initial crater morphometry for fresh craters in the crater modification model and the calculation of infill thickness based on crater morphometry. The crater morphometry has been thoroughly investigated in several different previous studies for Mars, with focuses on varying crater size and degradation state^16-21^. Most of these studies focus on scaling relationship for impact crater depths for different subsets of impact craters on Mars, in which three studies^16,17,20^ have presented both rim height and depth relationships to crater diameter for global and regional impact craters. These previous works also point out that the craters within the northern lowlands are significantly deeper than the highlands craters^17, 20, 21, 22^. These varying scaling relationships for crater morphometry in different geological provinces not only changes the depth-to-diameter ratio of fresh craters, but also directly control the removal of small craters in the resurfacing event via the initial rim height, as is shown in Supplementary Figure 4. The scaling relationships provided^16, 20^ for global impact craters underestimate the depth and rim height of observed freshest craters in Chryse Planitia (Supplementary Figure 4). On the other hand, we find that fresh craters in Acidalia Planitia^17^, the province adjacent to Chryse in the northern lowlands, are likely the most identical to the Chryse impact craters. These variations due to different initial crater geometries may shift the absolute values of the depth-to-diameter ratio (i.e. stronger target properties would increase depth-to-diameter ratio and vice versa) (Supplementary Figure 4). The bimodal distribution of depth-to-diameter ratio would not be affected by varying initial crater morphometry.

*Supplementary Note 6- Assumptions and input parameters of the crater accumulation model*

We use a crater accumulation model to estimate the effect of a widespread resurfacing event on crater statistics, assuming known impactor flux (i.e. crater production function and chronology^23,24^. We choose to use the same crater production and chronology functions to obtain the age of the unit from impact crater statistics (Figure 3a) and to calculate the resulting crater number densities as a function of time. Thus, the different choices of crater production and chronology functions do not change the resulting crater density from the accumulation model.

Here in detail, we show the different inputs to the crater accumulation and modification model (Supplementary Figure 5). We have found that apart from the thickness of the lava flow unit as discussed in the main text (Figure 3, Supplementary Figure 5a), the timing of the resurfacing event and adding a constant obliteration rate (Supplementary Figure 5b-c) would influence the resulting crater populations. Since the crater density is monotonically increasing without resurfacing and crater obliteration, the number of impact craters affected by the assumed resurfacing event is correlated to the timing of the event. It is expected that a late resurfacing event would result in a reduced number of fresh impact craters and relatively increased number of buried impact craters, but the resulting differences are not as significant as the variations of the thickness of infill. Here since we could observe a major resurfacing from the crater size-frequency diagram (Figure 3a), we find it unlikely that the major resurfacing of such scale would occur later than 3.7 Ga.

By adding a non-zero obliteration rate constant throughout the timeline, the number density of the buried impacts with depth to diameter ratio less than 0.05 would generally decrease since they would be completely removed through degradation and the depth of the pristine impact craters would be shallowed (Supplementary Figure 5). In comparison, the Amazonian erosion rate observed at the Pathfinder landing site is 0.01 m/Gyr, while this value could be higher in the Hesperian (0.1-10 m/Gyr) and Noachian (~10^2^-10^4^ m/Gyr)^25^. As such, the enhanced obliteration for small craters shown in the crater statistics (Figure 3a) indicates the obliteration within Chryse Planitia requires processes other than the moderate obliteration (predominantly by eolian erosion) in the Amazonian, probably related to secondary sedimentary or volcanic deposits within the Chryse Planitia.

**Supplementary Figure 1** Type examples of five different categories of degraded impact craters within the study region. The upper row is showing the elevation map from MOLA gridded topography data. The second row shows the day-time infrared imagery from THEMIS dataset. The scalebar in each image represents 10 km in length.


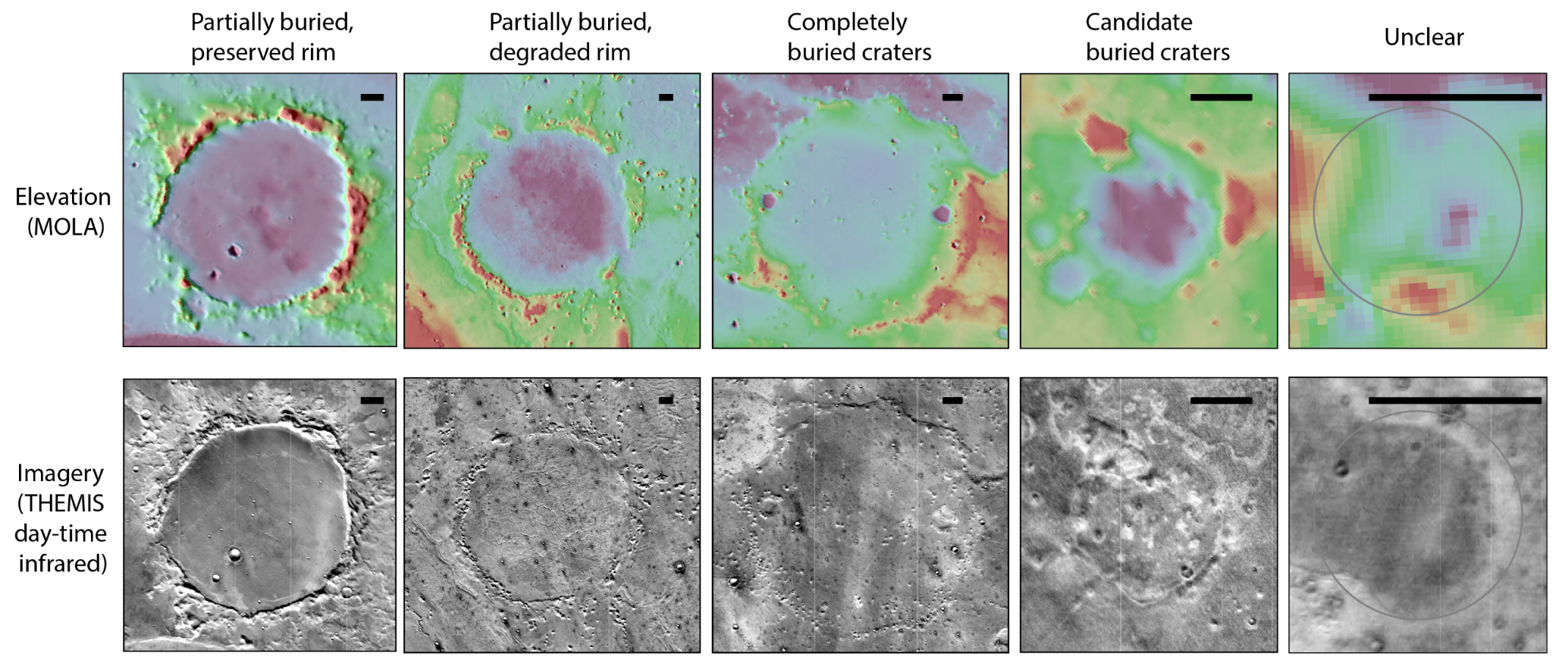


**
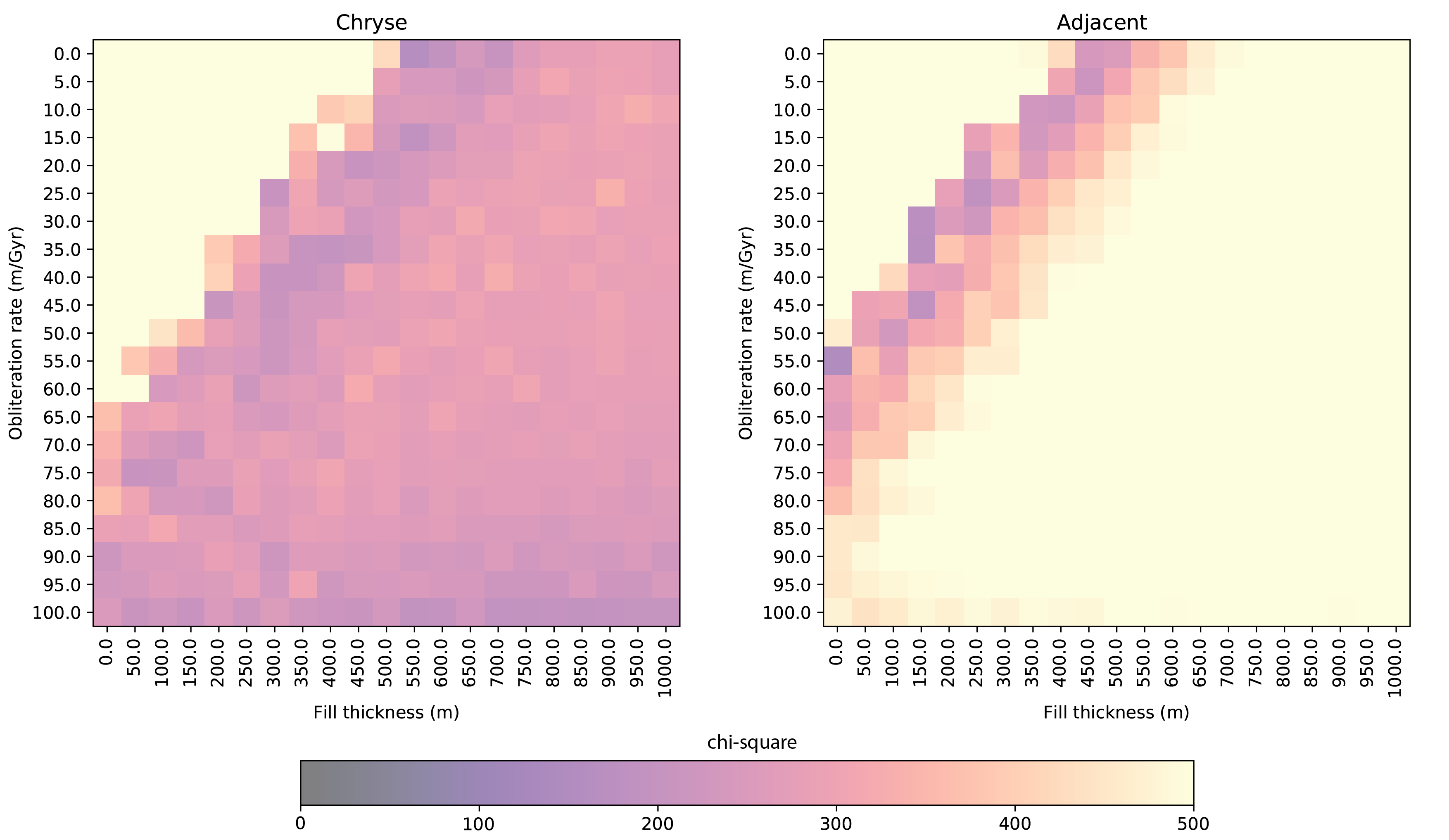
Supplementary Figure 2** Tests of the combination of a one-time resurfacing event at 3.7 Ga and a constant obliteration rate compared to both the Chryse and Adjacent unit. The colors represent chi-square statistics for the null hypothesis that the model and the observation generate the same population of impact craters for large, degraded craters (D>7km, d/D<0.04) in diameter bins.

**Supplementary Figure 3** Gravity anomaly profiles of Chryse unit and estimates of gravity anomaly for excess mass fill based on a plateau model. a) The gravity signal across the Chryse anomaly unit in different directions^26^. b) The corresponding MOLA elevation profiles across the Chryse anomaly unit as in a). c) The gravity anomaly expected with infill of dense materials using a simple plateau model. The density difference covers a range of realistic differences in material density for Mars. The inferred thickness of fill from observed buried craters is highlighted in the shaded area and corresponds to a small anomaly between 0 and 30 mgal.


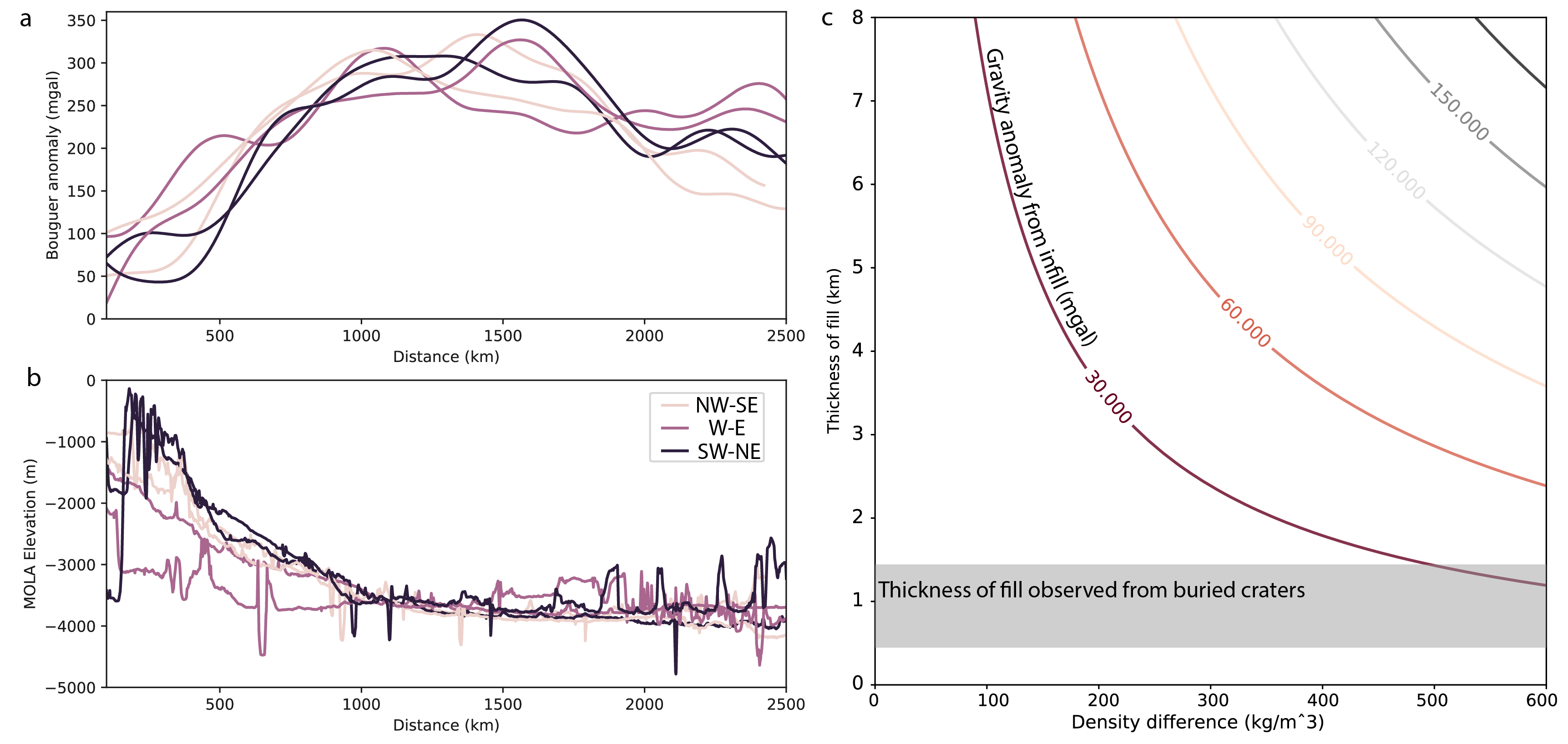


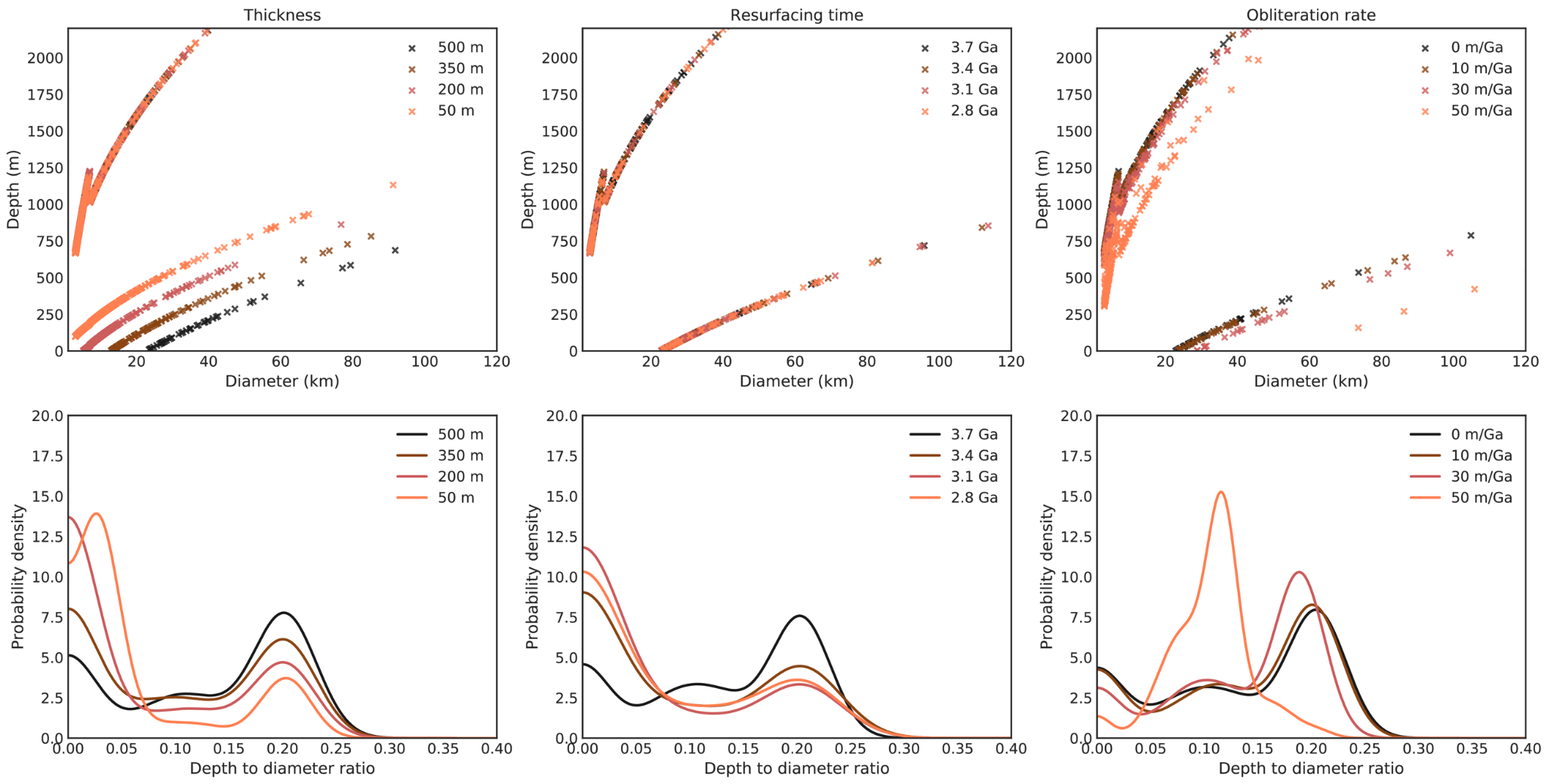
**Supplementary Figure 4** Crater accumulation and modification model with different initial crater morphometry. We show the results with 0 m/Gyr obliteration and a one-time infill event at 3.7 Ga using crater initial morphometry scaling provided in Ref. ^16, 20^ for global impact craters and from Ref. ^17^ for Acidalia Planitia craters. Global crater^16, 20^ which include shallower southern highlands craters, resulted
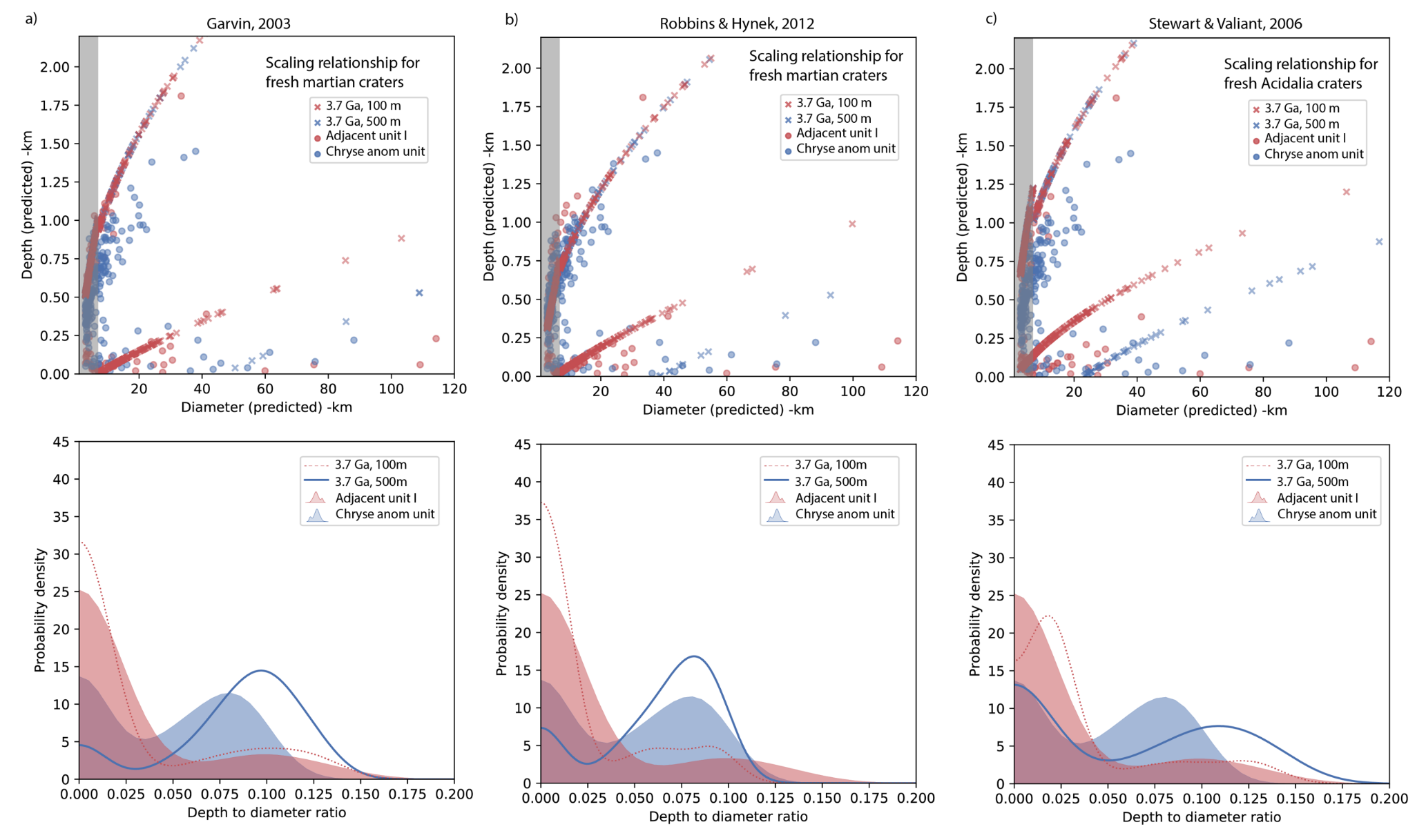
in smaller depths compared to the observed fresh craters in Chryse Planitia.


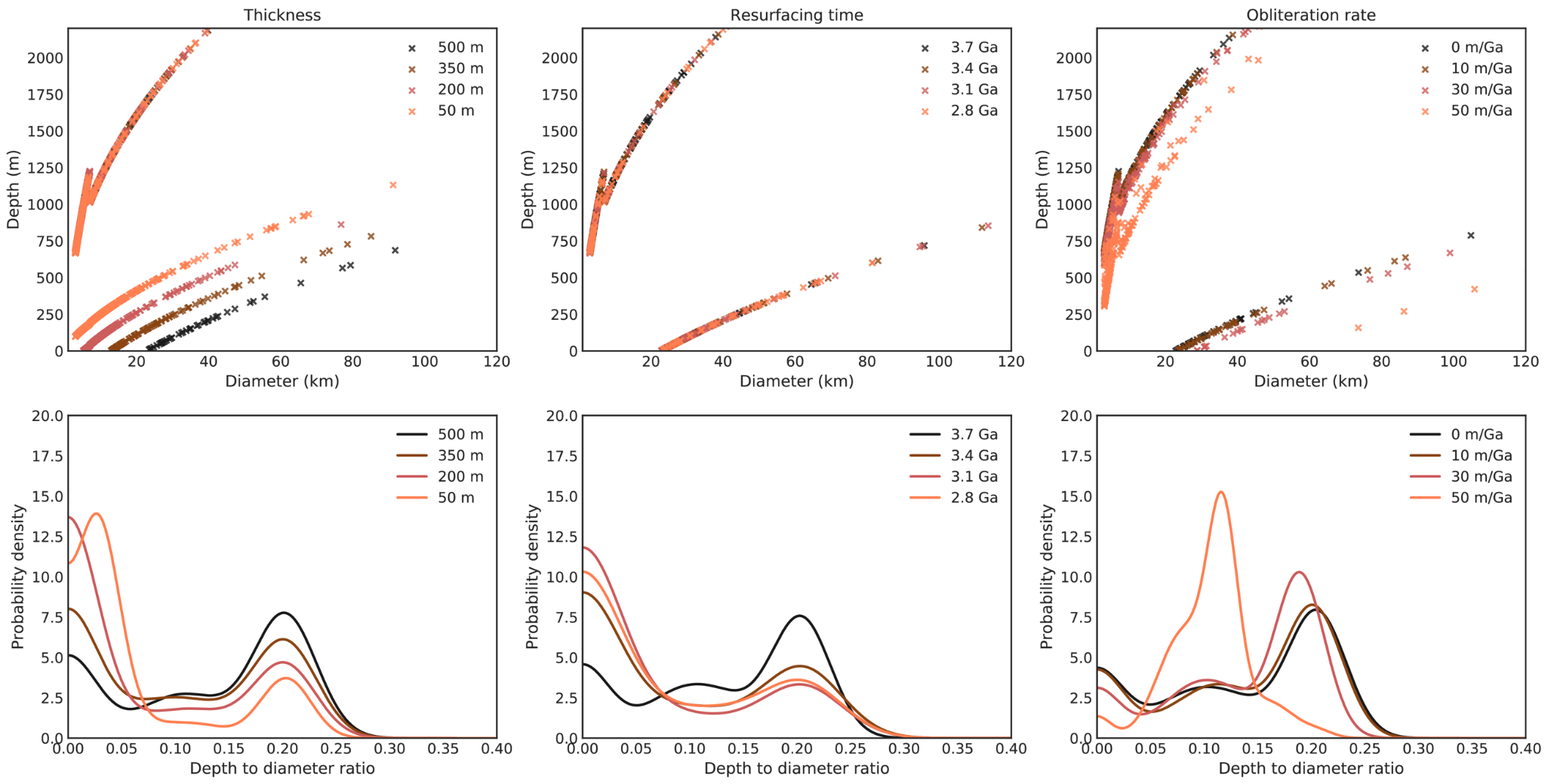
**Supplementary Figure 5** Crater accumulation and modification model with different thicknesses, resurface time and obliteration rate. We show here for one-time infill event, the crater population vary with the thickness of infill; the age of the resurfacing event, and the effect imposed constant obliteration rate (the title of each column indicates the changing variable in the plots of the same column). The black crosses and curve are showing the same base model, which is the blue scatter and curve in Figure 3b-c, assuming a resurfacing time at 3.7 Ga, with a layer thickness of 500m, and obliteration rate is 0 m/Ga.

**Supplementary Table 1:** Coefficient $a_{n}$ for the crater production function^23^. Here $a_{0}$ is calculated for a crater retention age of 1 Ga.

| $\boldsymbol{n}$ | $\boldsymbol{a}_{\boldsymbol{n}}$ | $\boldsymbol{n}$ | $\boldsymbol{a}_{\boldsymbol{n}}$ | $\boldsymbol{n}$ | $\boldsymbol{a}_{\boldsymbol{n}}$ | $\boldsymbol{n}$ | $\boldsymbol{a}_{\boldsymbol{n}}$ |
| --- | --- | --- | --- | --- | --- | --- | --- |
| _0_ | $-3.383677$ | _3_ | $0.7915374$ | _6_ | $1.015683\times{10}^{-1}$ | _9_ | $-4.753462\times{10}^{-3}$ |
| _1_ | $-3.197453$ | _4_ | $-0.4860814$ | _7_ | $6.755923\times{10}^{-2}$ | _10_ | $6.232845\times{10}^{-4}$ |
| _2_ | $1.256814$ | _5_ | $-0.3630098$ | _8_ | $-1.180639\times{10}^{-2}$ | _11_ | $5.805492\times{10}^{-5}$ |

**Supplementary Table 2:** Impact crater size-frequency statistics for Adjacent unit I (as shown in Figure 3a), with pseudo-log binning with a total area of $3.4\times{10}^{5}$ km^2^. Here D_min is the minimum diameter of each crater bin. F(D) is the number of impact craters. N_inc is the incremental crater density in #/km^2^. C(D) is the cumulative number of craters. N_cum is the cumulative crater density in #/km^2^.

| **D_min** | **F(D)** | **N_inc** | **Error** | **C(D)** | **N_cum** | **Error** |
| --- | --- | --- | --- | --- | --- | --- |
| 1 | 94 | 2.76E-04 | 2.84E-05 | 537 | 1.58E-03 | 6.80E-05 |
| 1.1 | 73 | 2.14E-04 | 2.51E-05 | 443 | 1.30E-03 | 6.17E-05 |
| 1.2 | 53 | 1.55E-04 | 2.14E-05 | 370 | 1.09E-03 | 5.64E-05 |
| 1.3 | 41 | 1.20E-04 | 1.88E-05 | 317 | 9.30E-04 | 5.22E-05 |
| 1.4 | 32 | 9.39E-05 | 1.66E-05 | 276 | 8.10E-04 | 4.87E-05 |
| 1.5 | 40 | 1.17E-04 | 1.86E-05 | 244 | 7.16E-04 | 4.58E-05 |
| 1.7 | 47 | 1.38E-04 | 2.01E-05 | 204 | 5.98E-04 | 4.19E-05 |
| 2 | 39 | 1.14E-04 | 1.83E-05 | 157 | 4.61E-04 | 3.68E-05 |
| 2.5 | 20 | 5.87E-05 | 1.31E-05 | 118 | 3.46E-04 | 3.19E-05 |
| 3 | 18 | 5.28E-05 | 1.24E-05 | 98 | 2.87E-04 | 2.90E-05 |
| 3.5 | 8 | 2.35E-05 | 8.30E-06 | 80 | 2.35E-04 | 2.62E-05 |
| 4 | 8 | 2.35E-05 | 8.30E-06 | 72 | 2.11E-04 | 2.49E-05 |
| 4.5 | 7 | 2.05E-05 | 7.76E-06 | 64 | 1.88E-04 | 2.35E-05 |
| 5 | 10 | 2.93E-05 | 9.27E-06 | 57 | 1.67E-04 | 2.21E-05 |
| 6 | 7 | 2.05E-05 | 7.76E-06 | 47 | 1.38E-04 | 2.01E-05 |
| 7 | 6 | 1.76E-05 | 7.18E-06 | 40 | 1.17E-04 | 1.86E-05 |
| 8 | 2 | 5.87E-06 | 4.15E-06 | 34 | 9.97E-05 | 1.71E-05 |
| 9 | 4 | 1.17E-05 | 5.87E-06 | 32 | 9.39E-05 | 1.66E-05 |
| 11 | 4 | 1.17E-05 | 5.87E-06 | 28 | 8.21E-05 | 1.55E-05 |
| 12 | 2 | 5.87E-06 | 4.15E-06 | 24 | 7.04E-05 | 1.44E-05 |
| 13 | 2 | 5.87E-06 | 4.15E-06 | 22 | 6.45E-05 | 1.38E-05 |
| 14 | 2 | 5.87E-06 | 4.15E-06 | 20 | 5.87E-05 | 1.31E-05 |
| 15 | 1 | 2.93E-06 | 2.93E-06 | 18 | 5.28E-05 | 1.24E-05 |
| 17 | 4 | 1.17E-05 | 5.87E-06 | 17 | 4.99E-05 | 1.21E-05 |
| 20 | 2 | 5.87E-06 | 4.15E-06 | 13 | 3.81E-05 | 1.06E-05 |
| 25 | 4 | 1.17E-05 | 5.87E-06 | 11 | 3.23E-05 | 9.73E-06 |
| 30 | 2 | 5.87E-06 | 4.15E-06 | 7 | 2.05E-05 | 7.76E-06 |
| 40 | 1 | 2.93E-06 | 2.93E-06 | 5 | 1.47E-05 | 6.56E-06 |
| 50 | 1 | 2.93E-06 | 2.93E-06 | 4 | 1.17E-05 | 5.87E-06 |
| 70 | 1 | 2.93E-06 | 2.93E-06 | 3 | 8.80E-06 | 5.08E-06 |
| 100 | 1 | 2.93E-06 | 2.93E-06 | 2 | 5.87E-06 | 4.15E-06 |
| 110 | 1 | 2.93E-06 | 2.93E-06 | 1 | 2.93E-06 | 2.93E-06 |

**Supplementary Table 3:** Impact crater size-frequency statistics for Chryse unit (as shown in Figure 3a), with pseudo-log binning with a total area of $7.90\times{10}^{5}$ km^2^. Here D_min is the minimum diameter of each crater bin. F(D) is the number of impact craters. N_inc is the incremental crater density in #/km^2^. C(D) is the cumulative number of craters. N_cum is the cumulative crater density in #/km^2^.

| **D_min** | **F(D)** | **N_inc** | **Error** | **C(D)** | **N_cum** | **Error** |
| --- | --- | --- | --- | --- | --- | --- |
| 1 | 321 | 4.07E-04 | 2.27E-05 | 1746 | 2.21E-03 | 5.29E-05 |
| 1.1 | 240 | 3.04E-04 | 1.96E-05 | 1425 | 1.81E-03 | 4.78E-05 |
| 1.2 | 179 | 2.27E-04 | 1.69E-05 | 1185 | 1.50E-03 | 4.36E-05 |
| 1.3 | 142 | 1.80E-04 | 1.51E-05 | 1006 | 1.27E-03 | 4.02E-05 |
| 1.4 | 109 | 1.38E-04 | 1.32E-05 | 864 | 1.09E-03 | 3.72E-05 |
| 1.5 | 157 | 1.99E-04 | 1.59E-05 | 755 | 9.56E-04 | 3.48E-05 |
| 1.7 | 128 | 1.62E-04 | 1.43E-05 | 598 | 7.57E-04 | 3.10E-05 |
| 2 | 126 | 1.60E-04 | 1.42E-05 | 470 | 5.95E-04 | 2.75E-05 |
| 2.5 | 79 | 1.00E-04 | 1.13E-05 | 344 | 4.36E-04 | 2.35E-05 |
| 3 | 52 | 6.59E-05 | 9.13E-06 | 265 | 3.36E-04 | 2.06E-05 |
| 3.5 | 30 | 3.80E-05 | 6.94E-06 | 213 | 2.70E-04 | 1.85E-05 |
| 4 | 22 | 2.79E-05 | 5.94E-06 | 183 | 2.32E-04 | 1.71E-05 |
| 4.5 | 25 | 3.17E-05 | 6.33E-06 | 161 | 2.04E-04 | 1.61E-05 |
| 5 | 22 | 2.79E-05 | 5.94E-06 | 136 | 1.72E-04 | 1.48E-05 |
| 6 | 19 | 2.41E-05 | 5.52E-06 | 114 | 1.44E-04 | 1.35E-05 |
| 7 | 20 | 2.53E-05 | 5.66E-06 | 95 | 1.20E-04 | 1.23E-05 |
| 8 | 14 | 1.77E-05 | 4.74E-06 | 75 | 9.50E-05 | 1.10E-05 |
| 9 | 12 | 1.52E-05 | 4.39E-06 | 61 | 7.73E-05 | 9.89E-06 |
| 10 | 8 | 1.01E-05 | 3.58E-06 | 49 | 6.21E-05 | 8.86E-06 |
| 11 | 7 | 8.86E-06 | 3.35E-06 | 41 | 5.19E-05 | 8.11E-06 |
| 12 | 2 | 2.53E-06 | 1.79E-06 | 34 | 4.31E-05 | 7.38E-06 |
| 13 | 2 | 2.53E-06 | 1.79E-06 | 32 | 4.05E-05 | 7.16E-06 |
| 14 | 3 | 3.80E-06 | 2.19E-06 | 30 | 3.80E-05 | 6.94E-06 |
| 15 | 3 | 3.80E-06 | 2.19E-06 | 27 | 3.42E-05 | 6.58E-06 |
| 17 | 5 | 6.33E-06 | 2.83E-06 | 24 | 3.04E-05 | 6.20E-06 |
| 20 | 6 | 7.60E-06 | 3.10E-06 | 19 | 2.41E-05 | 5.52E-06 |
| 25 | 1 | 1.27E-06 | 1.27E-06 | 13 | 1.65E-05 | 4.57E-06 |
| 30 | 1 | 1.27E-06 | 1.27E-06 | 12 | 1.52E-05 | 4.39E-06 |
| 35 | 3 | 3.80E-06 | 2.19E-06 | 11 | 1.39E-05 | 4.20E-06 |
| 40 | 3 | 3.80E-06 | 2.19E-06 | 8 | 1.01E-05 | 3.58E-06 |
| 45 | 1 | 1.27E-06 | 1.27E-06 | 5 | 6.33E-06 | 2.83E-06 |
| 50 | 1 | 1.27E-06 | 1.27E-06 | 4 | 5.07E-06 | 2.53E-06 |
| 60 | 1 | 1.27E-06 | 1.27E-06 | 3 | 3.80E-06 | 2.19E-06 |
| 70 | 1 | 1.27E-06 | 1.27E-06 | 2 | 2.53E-06 | 1.79E-06 |
| 80 | 1 | 1.27E-06 | 1.27E-06 | 1 | 1.27E-06 | 1.27E-06 |

**Supplementary References:**

1. Craddock, R. A., Maxwell, T. A. & Howard, A. D. Crater morphometry and modification in the Sinus Sabaeus and Margaritifer Sinus regions of Mars. *J. Geophys. Res. Planets*, 102, 13321–13340, (1997).
2. Forsberg-Taylor, N. K., Alan D. Howard & Craddock, R. A. Crater degradation in the Martian highlands: Morphometric analysis of the Sinus Sabaeus region and simulation modeling suggest fluvial processes. *J. Geophys. Res.*, 109, (2004).
3. Howard, A. D. Simulating the development of Martian highland landscapes through the interaction of impact cratering, fluvial erosion, and variable hydrologic forcing. *Geomorphology*, 91, 332–363, (2007).
4. Smith, M. R., Gillespie, A. R. & Montgomery, D. R. Effect of obliteration on crater-count chronologies for Martian surfaces. *Geophys. Res. Lett.*, 35, 2–7, (2008).
5. Craddock, R. A., Bandeira, L. & Howard, A. D. An Assessment of Regional Variations in Martian Modified Impact Crater Morphology. *J. Geophys. Res. Planets*, 123, 763–779, (2018).
6. Frey, H. V. Ancient lowlands on Mars. *Geophys. Res. Lett.*, *29*(10), 0–3. (2002).
7. Head, J. W., Kreslavsky, M. A., & Pratt, S. Northern lowlands of Mars: Evidence for widespread volcanic flooding and tectonic deformation in the Hesperian Period. *J. Geophys. Res.*, *107*(E1), 5003, (2002).
8. Buczkowski, D. L., Frey, H. V, Roark, J. H., & McGill, G. E. Buried impact craters: A topographic analysis of quasi‐circular depressions, Utopia Basin, Mars. *J. Geophys. Res.: Planets*, *110*(E3), (2005).
9. Buczkowski, D. L. Stealth quasi-circular depressions (sQCDs) in the northern lowlands of Mars. *J. Geophys. Res. Planets*, 112, 1–17, (2007).
10. Werner, S. C. Major aspects of the chronostratigraphy and geologic evolutionary history of Mars. Freien Universitat Berlin, (2005).
11. Bottke, W. F. & Andrews-Hanna, J. C. A post-accretionary lull in large impacts on early Mars. *Nat. Geosci.*, 10, 344–348, (2017).
12. Phillips, R. J., & Dvorak, J. The origin of lunar mascons: Analysis of the Bouguer gravity associated with Grimaldi. In *Multi-ring Basins, Proc. Lunar Planet. Sci.,* 91–104, (1981).
13. Melosh, H. J. et al., The Origin of Lunar Mascon Basins. *Science*, *340* (5), 1552–1556, (2013).
14. Arvidson, R. E. et al., Nature and Distribution of Surficial Deposits in Chryse Planitia and Vicinity, Mars. *J. Geophys. Res.,* 94, 1573–1587, (1989).
15. Tanaka, K. L., Robbins, S. J., Fortezzo, C. M., Skinner, J. A. & Hare, T. M. The digital global geologic map of Mars: Chronostratigraphic ages, topographic and crater morphologic characteristics, and updated resurfacing history. *Planet. Space Sci.*, 95, 11–24, (2014).
16. Garvin, J. B., Sakimoto, S. E. H. & Frawley, J. J. Craters on Mars: Global Geometric Properties from Gridded MOLA Topography. *Sxith Int. Conf.*, *Mars*, 7–9, (2003).
17. Stewart, S. T. & Valiant, G. J. Martian subsurface properties and crater formation processes inferred from fresh impact crater geometries. *Meteorit. Planet. Sci.*, 41, 1509–1537, (2006).
18. Boyce, J. M. & Garbeil, H. Geometric relationships of pristine Martian complex impact craters, and their implications to Mars geologic history. *Geophys. Res. Lett.*, 34, 1–5, (2007).
19. Whitehead, J., Grieve, R. A. F., Garvin, J. B. & Spray, J. G. The effects of crater degradation and target differences on the morphologies of Martian complex craters. *GSA Spec. Pap.*, 465, 67–80, (2010).
20. Robbins, S. J. & Hynek, B. M. A new global database of Mars impact craters ≥1 km: 2. Global crater properties and regional variations of the simple-to-complex transition diameter. *J. Geophys. Res. Planets*, 117, 1–21, (2012).
21. Tornabene, L. L. et al. A depth versus diameter scaling relationship for the best-preserved melt-bearing complex craters on Mars. *Icarus*, 299, 68–83, (2018).
22. Boyce, J. M., Mouginis-Mark, P., Garbeil, H. & Tornabene, L. L. Deep impact craters in the Isidis and southwestern Utopia Planitia regions of Mars: High target material strength as a possible cause. *Geophys. Res. Lett.* 33, 2–5, (2006).
23. Ivanov, B. A. Mars/Moon cratering rate ratio estimates. in Cratering chronology and Evolution of Mars, 96, 87–104, (2001).
24. Hartmann, W. K. & Neukum, G. Cratering chronology and the evolution of mars. *Space Sci. Rev.*, 96, 165–194, (2001).
25. Golombek, M. P. & Bridges, N. T. Erosion rates on Mars and implications for climate change: Constraints from the Pathfinder landing site. *J. Geophys. Res.*, 105, 1841–1853, (2000).
26. Genova, A. et al. Seasonal and static gravity field of Mars from MGS, Mars Odyssey and MRO radio science. *Icarus*, 272, 228–245, (2016).
